# Systematic review and meta-analysis of cryopreserved bovine sperm assessment: harnessing imaging flow cytometry for multi-parametric analysis

**DOI:** 10.3389/fvets.2024.1371586

**Published:** 2024-04-24

**Authors:** Anel Umirbaeva, Andrey Kurenkov, Aizhan Makhanbetova, Bolat Seisenov, Ivan A. Vorobjev, Natasha S. Barteneva

**Affiliations:** ^1^Department of Biology, School of Sciences and Humanities, Nazarbayev University, Astana, Kazakhstan; ^2^Department of Computer Sciences, School of Engineering and Digital Sciences, Nazarbayev University, Astana, Kazakhstan; ^3^JSC “Republican Center of Breeding in Livestock” “Asyl-Tulik”, Kosshi, Kazakhstan

**Keywords:** sperm, cryopreservation, meta-analysis, flow cytometry, bovine semen quality, imaging flow cytometry, tetramethylrhodamine methyl ester, image-based sorting

## Abstract

Cryopreservation of sperm is an essential technique in assisted reproduction in cattle. The objective of the study was to systematically review and synthesize the literature on bull semen quality evaluation based on the comparison of morphological and metabolic parameters of cryopreserved bovine spermatozoa such as DNA integrity, mitochondrial status, plasma membrane alterations, total motility, and morphology (% of abnormal cells). The electronic databases PubMed, Web of Sciences, Scopus, and Google Scholar were searched up to December 2023. Studies and references were included if they reported the following parameters: DNA integrity, mitochondrial status, plasma membrane alterations, total motility, and morphological aberrations (% of abnormal cells) for conventional cryopreserved bovine spermatozoa. After an electronic search, out of 1,526 original studies, only 40 were included in the meta-analysis. Standardized mean differences (SMD) with 95% confidence intervals were estimated for the chosen studies, and a meta-analysis was performed using a random effects model. The tau-squared (tau^2^) and inconsistency index (*I*^2^) quantified heterogeneity among different studies. The regression analysis for the evaluated parameters showed a positive correlation between mitochondrial membrane potential (MMP), total motility, and abnormal morphology and a negative correlation between DNA fragmentation index (DFI) and total motility and MMP. Moreover, subgroup analysis demonstrated similar associations for dairy and non-dairy bull breeds, albeit with lower *I*^2^ values. The presence of publication bias was confirmed by Egger’s test, except for the MMP parameter. A multi-parametric analysis of morphological and metabolic parameters can address the existing limitations of cryopreserved bovine spermatozoa quality assessment. Combining imaging flow cytometry (IFC) with standardization of sperm pre-processing and optimization of the experimental protocols may help to differentiate sperm from cellular debris and cytoplasmic droplets of similar size and alleviate limitations demonstrated by conventional sperm analysis.

## 1 Introduction

There is an urgent need to increase the fertility of livestock species and improve the efficiency of food-producing animals to overcome the growing demand for food and animal protein access. Cryopreservation of sperm is an essential aspect of breeding in agriculture (1–4), allowing the storage of selected gametes at liquid nitrogen temperature and the preservation of the genetic material of the cells with their structural and functional integrity (5, 6). Artificial insemination relies on frozen-thawed bull semen and has the greatest impact on the genetic breeding of cattle (7). The sperm quality assessment is crucial for the success of artificial insemination and breeding in cattle (8). Despite early reassurances that this temperature arrests all metabolic processes and sperm quality would not be affected (9), concerns have recently arisen regarding the functionality and quality of cryopreserved sperm after long-term storage (1, 10). In general, 40–50% of the sperm does not survive the cryopreservation procedure (6). Post-thawed spermatozoa, particularly in cattle, are very sensitive to temperature changes (11) and rapidly decline in viability after post-thawing (12–15). However, conventional sperm analysis is time-consuming, and due to the absence of standardized protocol and subjectivity of the microscopic assessment, results from multiple laboratories are highly variable (16–21). The flow cytometric approach has the advantage of evaluating multiple cellular features of spermatozoa; however, it does not allow evaluation of the morphological parameters (22–24).

As of today, there is no single *in vitro* sperm quality assessment test that allows accurate evaluation of the quality of sperm and prediction of sperm fertilizing potential (25). The most promising tests available include sperm viability assessment. With the rise of new technologies such as computer-assisted sperm analysis (CASA) systems and flow cytometry, subsequent studies started to pay closer attention to combining the morphological features and metabolic parameters of sperm cells to assess sperm quality for proper fertilization. Changes in the plasma membrane integrity, mitochondrial potential, chromosome integrity, acrosome, and axoneme structures (26–29) may decrease sperm viability and lead to low fertility (30). During past decades, meta-analyses were used to investigate the possible associations between morphological sperm parameters and the overall fertility state of bulls (31, 32).

This systematic review and meta-analysis aimed to compare studies describing the major morphological and metabolic parameters of cryopreserved bovine spermatozoa to evaluate their relationship to each other and state after cryodamage. We are also discussing imaging flow cytometry as a possible hybrid technology for multi-parametric analysis, and providing an example of multi-parametric morphological and functional analysis of bovine sperm.

## 2 Materials and methods

### 2.1 Search strategy

An electronic search of Google Scholar, MEDLINE (PubMed), Scopus, and ISI Web of Science databases was performed up to December 1, 2023. The combination of the following keywords and search terms were used: “bull,” “semen evaluation,” “bovine sperm” AND “parameter,” “sperm,” “DNA fragmentation,” “morphology,” “mitochondria,” “viability” AND “assessment” OR “cryopreservation,” and “thawed.” The search was conducted by two independent reviewers, initially resulting in 1526 articles. The search process and results are depicted in the flow diagram. Additionally, the reviewers manually checked reference lists of relevant articles and reviews in search of potentially eligible studies.

### 2.2 Inclusion and exclusion criteria

The articles were excluded and included in the first case based on their titles and abstract contents. In the second case, full-text articles were indexed, and criteria targeted a paper’s title and complete contents. First, full versions of the articles went through the exclusion criteria, which included (1) non-bovine sperm, (2) no required parameters, (3) pregnancy rates research, (4) evaluation of extender effect, (5) cryoprotectant efficiency, (6) non-cryopreserved semen, (7) cell signaling research, (8) proteomic or lipidomic analysis. Additionally, we excluded preprints and data from subgroups of treated sperm from studies that had measured post-thawing parameters after applying some treatment. Then, the remaining articles were checked according to inclusion criteria, which required (1) target parameters to have mean and standard deviation data, (2) detailed quantitative data on viability and some of the next parameters: morphology, mitochondrial potential, motility and DNA fragmentation, (3) sperm sample origin, (4) data on hours after thawing, and (5) sample sizes. Selected articles should have at least one of the parameters studied for bovine semen quality along with the viability or, in some cases, given as the plasma and acrosomal membrane integrity or plasma membrane integrity. The sample size was taken as the number of bulls whose semen was evaluated, and the mean and standard deviation values should be given for all the bulls, not for their ejaculates or batches. When the samples are analyzed and divided into groups (fertility differences, age, bulls’ specialization, etc.), each subgroup should be used for the analysis as it is without further mathematical calculations for deriving common values for the groups. These measurements were undertaken to avoid biased and erroneous evaluations of collected data.

### 2.3 Data collection and data items

Final data were obtained from all the eligible studies, including the author’s name, year of publication, sample size, bull breed, study design, description of the parameters, and measurable methods. The search results were extracted from scientific databases and uploaded in EndNote X9. Measurements of post-thawing sperm parameters [motility, DNA fragmentation index (DFI), viability, morphology, mitochondrial membrane potential (MMP)] were extracted into the data table and grouped by their fertility rates, breeds, and locations. The second reviewer downloaded full-text articles of all search results and used system and software indexation to apply exclusion and inclusion criteria. The same parameters were then transferred to the data table. Additional information on the studies’ characteristics can be found in Supplementary Tables S1, S2.

### 2.4 Statistics and meta-analysis

Sperm viability was taken as a control parameter since it is one of the important fertility factors, presenting the number of live cells. The viability was estimated to have a mean of 51.720 throughout 59 groups within the studies that were included in the meta-analysis. The minimum and maximum values were 26.13 and 84.43, respectively, with a 95% confidence interval (CD) from 48.466 to 54.794 inclusively. In several analyzed studies, results were given as mean and standard error (SE) for each of the bulls or their ejaculates (presented as straws or batches). Therefore, we calculated the standard deviation (SD) and the sample size (n) according to the pooled standard deviation formula (33). Data parameters were extracted from the source csv file and exported to Pandas DataFrame. Pandas v.2.0 and Matplotlib v.3.7.2 were used to study the data and check for preliminary correlations and outliers. Further, DataFrame columns with desired parameters were transformed into NumPy arrays to fit the data using matrix operations, yielding linear regression lines and planes describing the variables’ relationship.

Heterogeneity between studies was evaluated using *Q* test and *I*^2^ statistics (34) and tau squared. The latest was estimated through the restricted maximum likelihood procedure, which shows particular robustness in the random effects model. In each category with more than five high-quality studies, subgroup analysis and univariate regression were conducted to explore sources of heterogeneity. As *I*^2^ > 50% was expected between studies, all analyses were performed using the random effect model (35). As a part of the statistical analysis, we conducted a traditional and multiple regression analysis (36). Both were estimated in Python v.3.8 using the NumPy v.1.22.0 and Seaborn library v.012, including plotting and scientific calculations. IBM SPSS vs. 29.0 (IBM, United States) and Python Meta package with supporting libraries for data analysis (Pandas, NumPy) were used to analyze the available data, including linear regression, multiple linear regression, estimation of publication bias, and subgroup analysis. In the calculations, we used weighting techniques to approximate the individual effects of each study.

### 2.5 Sensitivity and subgroup analysis

The sensitivity analysis was performed by using a leave-one-out method and re-evaluating the effect sizes of the studies. Then, outlier studies whose confidence interval values did not coincide with the confidence interval of pooled effect sizes were removed. For further investigation of heterogeneity within studies, subgroup analysis was conducted based on fertility and the origin of breed data. Cattle breeds may significantly differ in metabolic parameters (37). According to the source of breeds, bulls mainly included dairy and non-dairy groups. The dairy breeds were Holstein, Holstein Friesian, Estonian Holstein, Brown Swiss, Swedish white and red, Sahiwal, Finnish Ayrshire, and Norwegian Red. The non-dairy group included beef bulls of the following breeds: Blonde d’Acquitaine, Limousin, Red Angus, Tropical Montana, Pinzgau, Japanese Black, Belgian Blue, Hereford, and Charolais; dual-purpose included Simmental, Senepol, Nellore, and *Bos Indicus* cattle.

### 2.6 Publication bias

Given the difficulty of correcting and detecting publication bias, we assessed data by measuring funnel plot asymmetry. Funnel plots of primary outcomes were visually and formally evaluated with Egger’s test (38) with *p* < 0.05, indicating significant publication bias.

### 2.7 Imaging flow cytometry of bovine sperm

Semen was collected from Kazakh White-headed breed bulls from qualifying ejaculates (motility >60%, evaluated with CEROS II sperm-analysis CASA system (Hamilton Thorne, United States); concentration > 0.5 billion/mL, evaluated by photometry using FEK-M (RF) instrument; volume > 1 mL), diluted with OptiXcell^®^ (IMV Technologies, France). Diluted semen was cooled, equilibrated, and filled in 0.5 mL straws for cryopreservation in liquid nitrogen. OptiXcell^®^ is advantageous compared to other yolk-free commercial sperm extenders (39). Chemicals, including tetramethylrhodamine methyl ester (TMRM), DMSO, and Hoechst 33342 dye, were purchased from Sigma-Aldrich (United States).

Imaging flow cytometry analysis was performed using Imagestream X Mark II instrument (Amnis-Cytek, United States) equipped with combination of lasers (405, 488, 561, and 640 nm) and objectives as described elsewhere (40). Shortly, a semen sample straw was transferred from −86°C and thawed quickly in a water bath at 37°C as preparation for further analysis. TMRM (Sigma-Aldrich, United States) was kept as a stock solution in dimethyl sulfoxide (DMSO) at 20 mmol/L, aliquoted and stored at −20°C. When the sample was in the water bath, TMRM was added to the sperm at the final concentration of 5–100 ng/mL, and the sample was incubated in the dark for 15 min at room temperature. Additional staining with Hoechst 33342 dye (concentration 10–100 nm) was done before the acquisition of the sperm sample with Imagestream. Debri and multiple events were excluded by sequential gating with IDEAS (Amnis-Cytek, United States) software.

## 3 Results

### 3.1 Results of search

**Figure 1:**
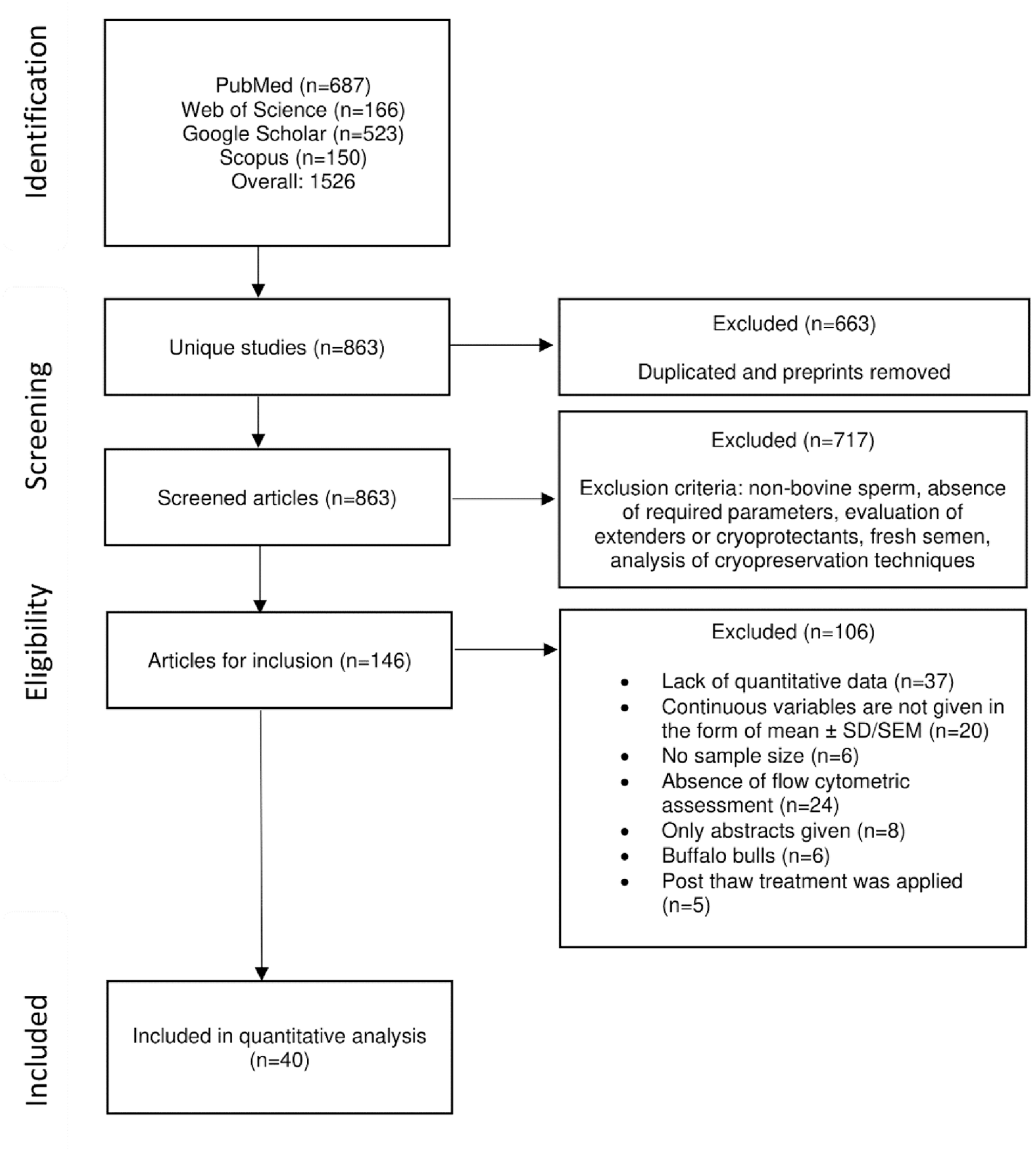
Flow diagram of study selection.

The detailed process of the data literature search is depicted in Figure 1. Overall, 1,526 articles were identified as a result of a web search. After the duplicated removal of duplicates, only 863 studies were considered unique. Finally, exclusion and inclusion criteria were applied for a more thorough investigation. Eventually, 40 studies were found eligible and were included in our study.

### 3.2 Studies characteristics

In total, sperm samples obtained from 972 bulls are included in this meta-analysis from 40 original studies. The studies mainly belonged to three breed origin groups: Northern Europe, Western Europe, and others. Breeds included in the last group are Nellore (beef breed; India), Pinzgau (a triple-purpose breed, raised for meat, milk, and draught use from Austria-i.e., Western Europe), Tropical Montana (beef cattle, Brazil), Senepol (beef and dairy), American Brahman (beef cattle), and unmentioned ones. Moreover, the studies were characterized by their sperm quality assessment methods: flow cytometry and microscopy. Detailed information on methods and stains used for sperm evaluation is shown in Supplementary Table S2.

### 3.3 Pooled effect sizes

Overall, data was collected for the following sperm quality parameters: viability (control), abnormal morphology, total motility, DNA fragmentation index (DFI), and mitochondrial membrane potential (MMP). Heterogeneity was assessed using the *I*^2^, tau^2^, and H-statistics. Tau^2^ and *I*^2^ are our primary metrics in the assessment of heterogeneity. *I*^2^ represents the percentage of total variation across studies due to heterogeneity (41). Tau^2^ is the estimate of the variance of the effect sizes. A larger value indicates greater heterogeneity, and the greater deviation from 0 suggests the use of a random effects model and greater standard deviation in the true effect sizes. H is the square root of the tau^2^ value divided by its degree of freedom (34, 42). For all parameters analyzed, the *I*^2^ values were >78.1%, and tau^2^ ranged from 0.453 to 12.491 (Table 1), which required using the random effects model.

**Table 1:** Summary of the heterogeneity assessment and sensitivity analysis.

Effect size					Heterogeneity	
Parameter		Pooled SMD	95% CI	*p*-value	*I*^2^ (%)	Tau^2^
DFI	Original	−5.699	[−7.025; −4.374]	<0.01	95.0	12.491
	No outliers	−4.103	[−4.873; −3.334]	<0.01	87.2	3.220
MMP	Original	−0.489	[−1.173; 0.195]	0.160	95.0	3.720
	No outliers	−0.401	[−0.707; −0.095]	<0.01	71.0	0.453
Morphology	Original	−3.212	[−4.052; −2.285]	<0.01	90.8	3.781
	No outliers	−2.615	[−3.250; −2.002]	<0.01	83.3	1.822
Motility	Original	0.359	[−0.750; 1.493]	<0.01	97.9	12.162
	No outliers	0.571	[0.247; 0.936]	<0.01	77.8	0.769

**Figure 2:**
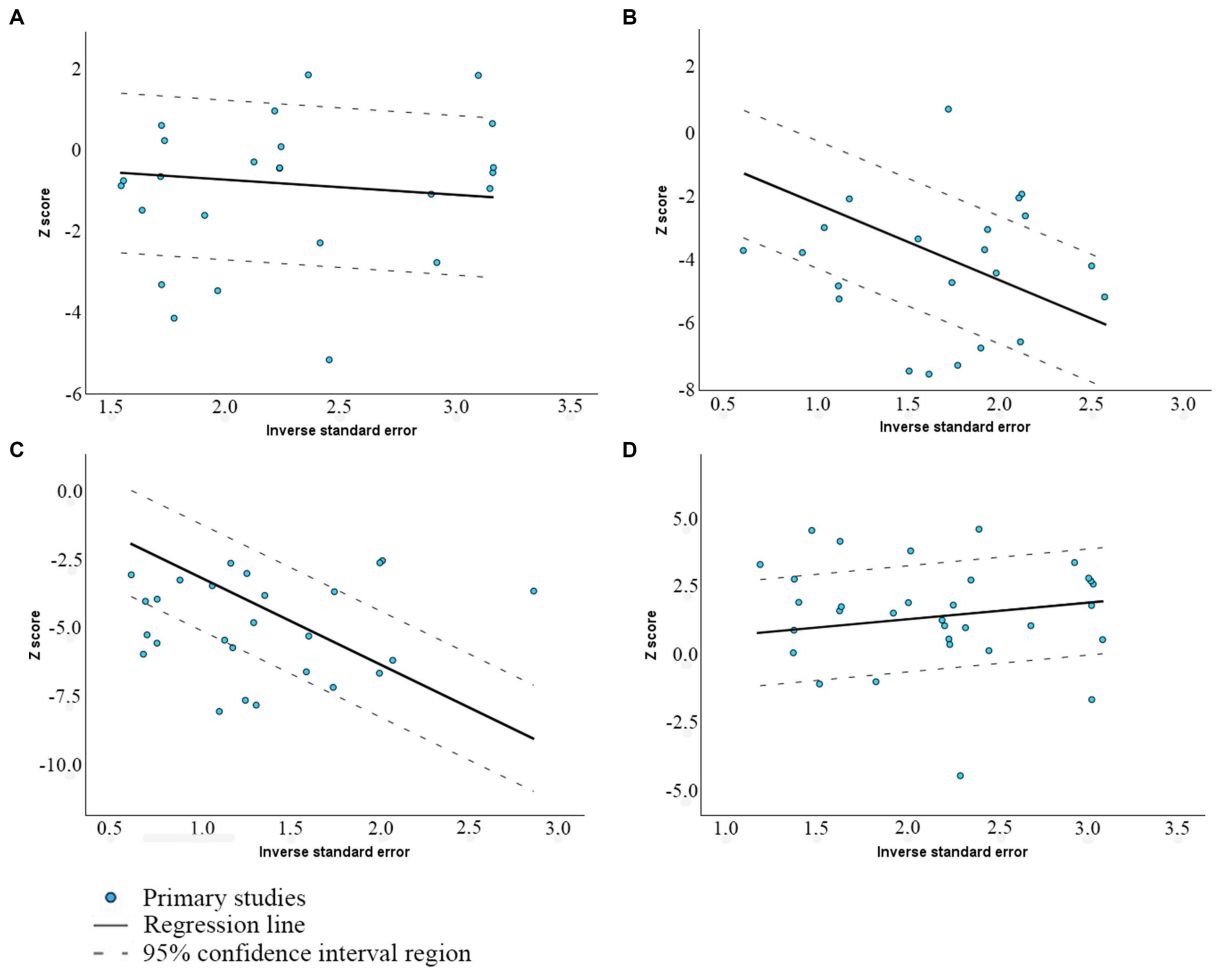
Galbraith plot of the association between viability and **(A)** MMP; **(B)** Abnormal morphology; **(C)** DFI; and **(D)** Total motility.

Figure 2 presents Galbraith plots of this meta-analysis, illustrating the confidence intervals (dotted lines) of various studies alongside the linear regression line, allowing for the identification of potential outliers. MMP and total motility regression lines have neutral to moderate positive slopes, suggesting that the source of the heterogeneity might come from differences at the stages of sampling, analysis, or random variations, which attests to homogeneity. The Galbraith plots for abnormal morphology and DFI show a negative trend, suggesting insufficiency of sample sizes in studies and significant standard error, increasing as the study size decreases.

### 3.4 Sensitivity analysis

The leave-one-out method identified studies that significantly influence the effect size estimates. Outliers were selected by both their individual and pooled confidence intervals. Removal of the outliers resulted in a decrease in the heterogeneity without affecting the overall results; however, it left the level of heterogeneity still high (>50%). Original effect sizes and ones with removed outliers are depicted in Table 1. Corresponding graphs for the studies without outliers are provided in Supplementary Figure S1.

### 3.5 Subgroup analysis

A detailed subgroup analysis was performed to study the sources of heterogeneity observed in the meta-analysis. The subgroup analysis was delineated by breed type, Dairy or Beef/Mix/Dual, which provides a more specific look into the variances across different genetic backgrounds. The effect sizes and heterogeneity measures for each parameter and subgroup are comprehensively presented in Table 2. The data indicates differences between the Dairy and Non-Dairy (Beef/Mix/Dual) subgroups across all parameters, with notably distinct effect sizes and levels of heterogeneity, explainable by smaller study sizes.

**Table 2:** Summary of the subgroup analysis.

Parameter	Subgroup	Effect size			Heterogeneity	
		*k*	SMD [95% CI]	*p*-value	*I*^2^ (%)	Tau^2^
DFI	Dairy	19	−3.978 [−4.942; −3.013]	<0.01	79.1	2.455
	Beef/Mix/Dual	7	−4.473 [−5.619; −3.328]	<0.01	88.0	3.103
MMP	Dairy	23	−0.520 [−0.874; −0.166]	<0.01	79.9	0.347
	Beef/Mix/Dual	3	−0.125 [−0.643; −0.394]	<0.01	91.5	2.566
Morphology	Dairy	16	−2.780 [−3.502; −2.057]	<0.01	78.7	0.840
	Beef/Mix/Dual	7	−2.290 [−3.836; −0.722]	<0.01	94.8	3.810
Motility	Dairy	22	0.742 [0.526; 0.959]	<0.01	72.4	1.123
	Beef/Mix/Dual	14	0.342 [−0.566; 1.309]	0.41	91.0	4.427

### 3.6 Linear regression analysis

**Figure 3:**
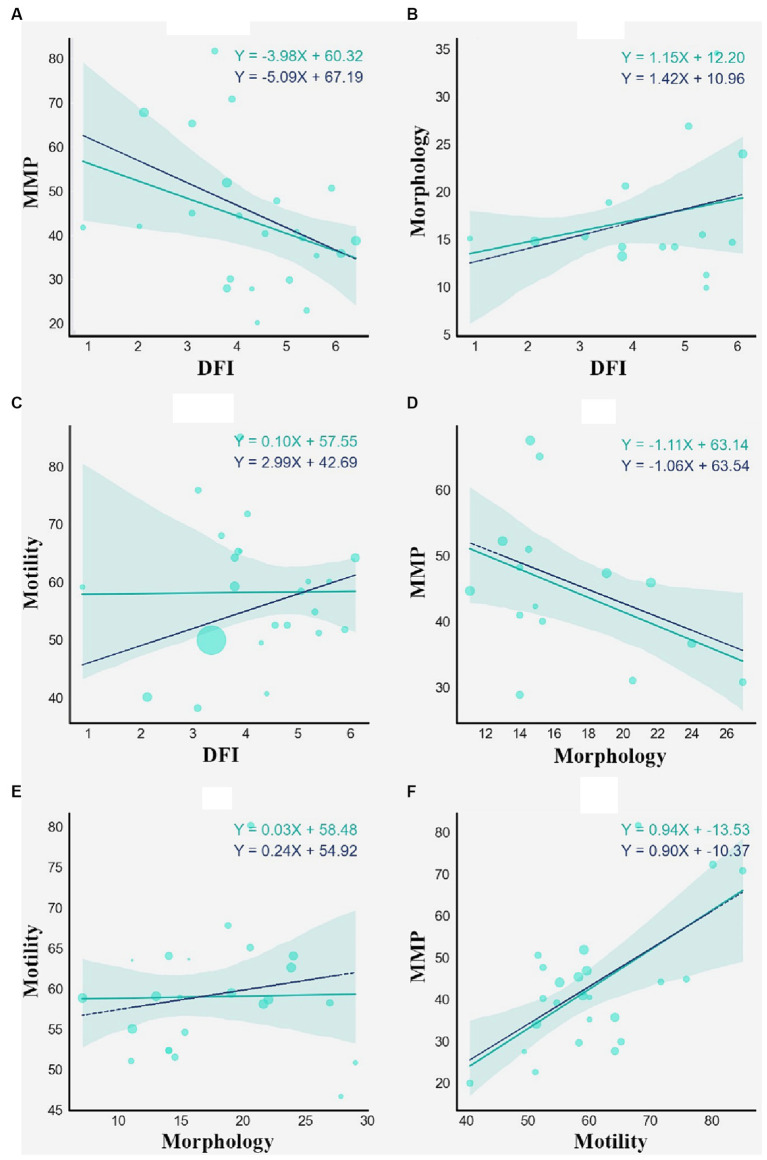
Weighted linear regression of DFI and MMP **(A)**, DFI and Morphology **(B)**, DFI and Motility **(C)**, Morphology and MMP **(D)**, Morphology and Motility **(E)**, Motility and MMP **(F)**. The cyan solid regression line represents non-weighted linear regression. The blue dashed line represents weighted linear regression, each weight associated with the sample size of a study.

The relationship between the individual parameters was also evaluated and illustrated via linear regression plots. We used it as the first stage in assessing the value and influence of independent parameters on each other. DFI-MMP, Motility-MMP, and Morphology-MMP models yield statistically significant results, suggesting the presence of general trends even in high heterogeneity settings. Further, multiple regression provides better intuition on the relation between the parameters. The plots in Figure 3 show the association between independent parameters: MMP, morphology, DFI, and total motility. Data points are represented by mean values of parameters in %. The statistical significance of the models was evaluated using F-statistics and *R*^2^ values (Table 3).

**Table 3:** Statistical results of linear regression modeling.

Plot	*k*	Weighted		Non-weighted	
		*R* ^2^	F-statistics	*R* ^2^	F-statistics
DFI-MMP	23	0.311	5.614	0.342	2.911
DFI-Morphology	16	0.117	1.847	0.064	0.964
DFI-Motility	23	0.099	2.309	0.001	0.002
Morphology-MMP	18	0.374	4.742	0.286	3.964
Morphology-Motility	22	0.032	0.731	0.001	0.001
Motility-MMP	25	0.378	13.96	0.409	15.93

### 3.7 Multiple regression analysis

**Figure 4:**
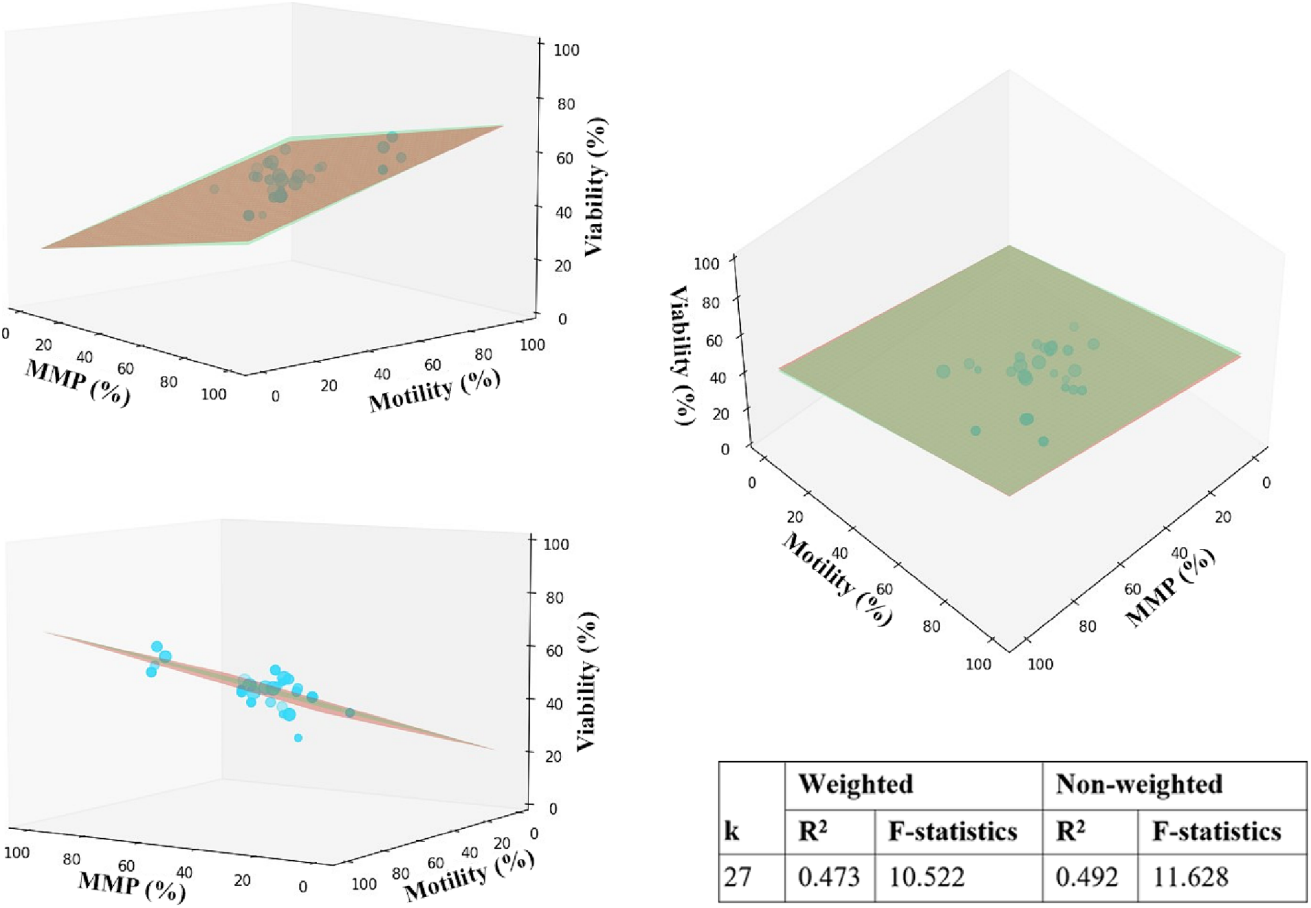
Multiple linear regression analysis for motility, MMP, and viability. The 2D red and green planes represent unweighted and weighted models, accordingly. Data points are represented by mean values of parameters in %, scaled in size according to the corresponding sample size.

Regression analyses were conducted to identify the relationship between the independent parameters and factors influencing the heterogeneity of studies. Observations from a meta-analysis suggest that MMP and motility data are less biased and might provide additional insights into the relationship between viability and fertility. We specified one dependent variable (viability) and two independent variables (MMP, motility) to model the relationship in 3D space. It is expected that the viability will be directly proportional to MMP and motility, and multiple regression analysis helped to confirm the assumptions in the presence of strong variations across studies. We selected studies with data for required variables and estimated the regression plane using matrix operations with NumPy and Scipy. The *Z*-axis of the 2D plane was optimized using a least squares solution, and then the plane was plotted using Matplotlib. As shown in Figure 4, motility has a slightly stronger effect on viability since the plane’s slope is steeper than MMP. MMP also positively correlates with viability.

### 3.8 Publication bias

**Figure 5:**
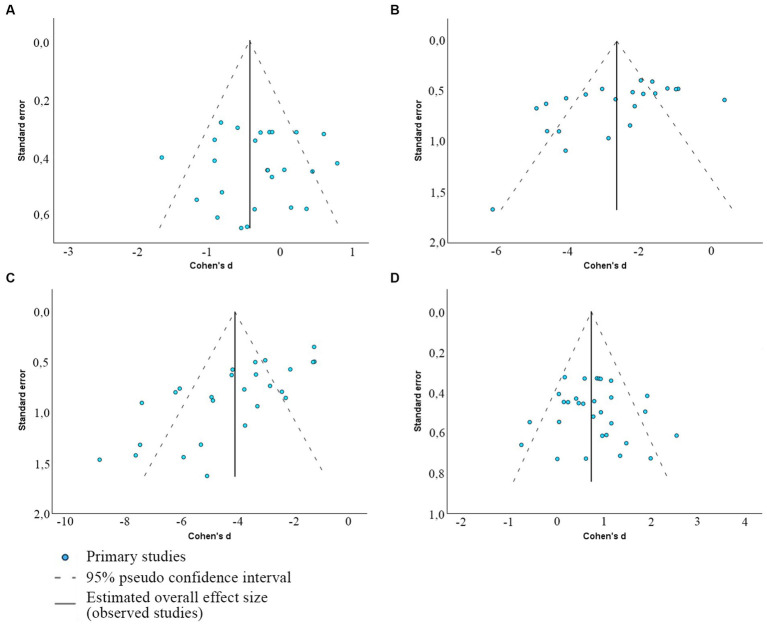
Funnel plots depicting the presence of publication bias between included articles that determine the association between viability and MMP **(A)**, morphology **(B)**, DFI **(C)**, and total sperm motility **(D)**.

Egger’s regression-based test for publication bias yielded significant results for several sperm quality parameters, indicating potential bias (*p* < 0.01 for DFI and morphology; *p* = 0.012 for motility) (Table 4). However, for MMP, results suggest a lack of publication bias (*p* = 0.088). These interpretations are visually supported by the funnel plots with outliers presented in Figure 5 (and Supplementary Figure S2—with outliers) depicting the spread and symmetry of the studies’ effect sizes against their precision.

**Table 4:** Results of Egger’s test for sperm quality parameters.

Parameter	Intercept	95% Confidence interval	*t*	*p*-value
DFI	−1.449	[−2.235; −0.663]	−2.213	<0.001
MMP	0.449	[−0.930; 1.677]	1.390	0.088
Morphology	−0.996	[−2.102; −0.062]	−2.980	<0.001
Motility	1.769	[−0.024; 3.598]	3.102	0.012

### 3.9 Imaging flow cytometry analysis of thawed bull sperm

**Figure 6:**
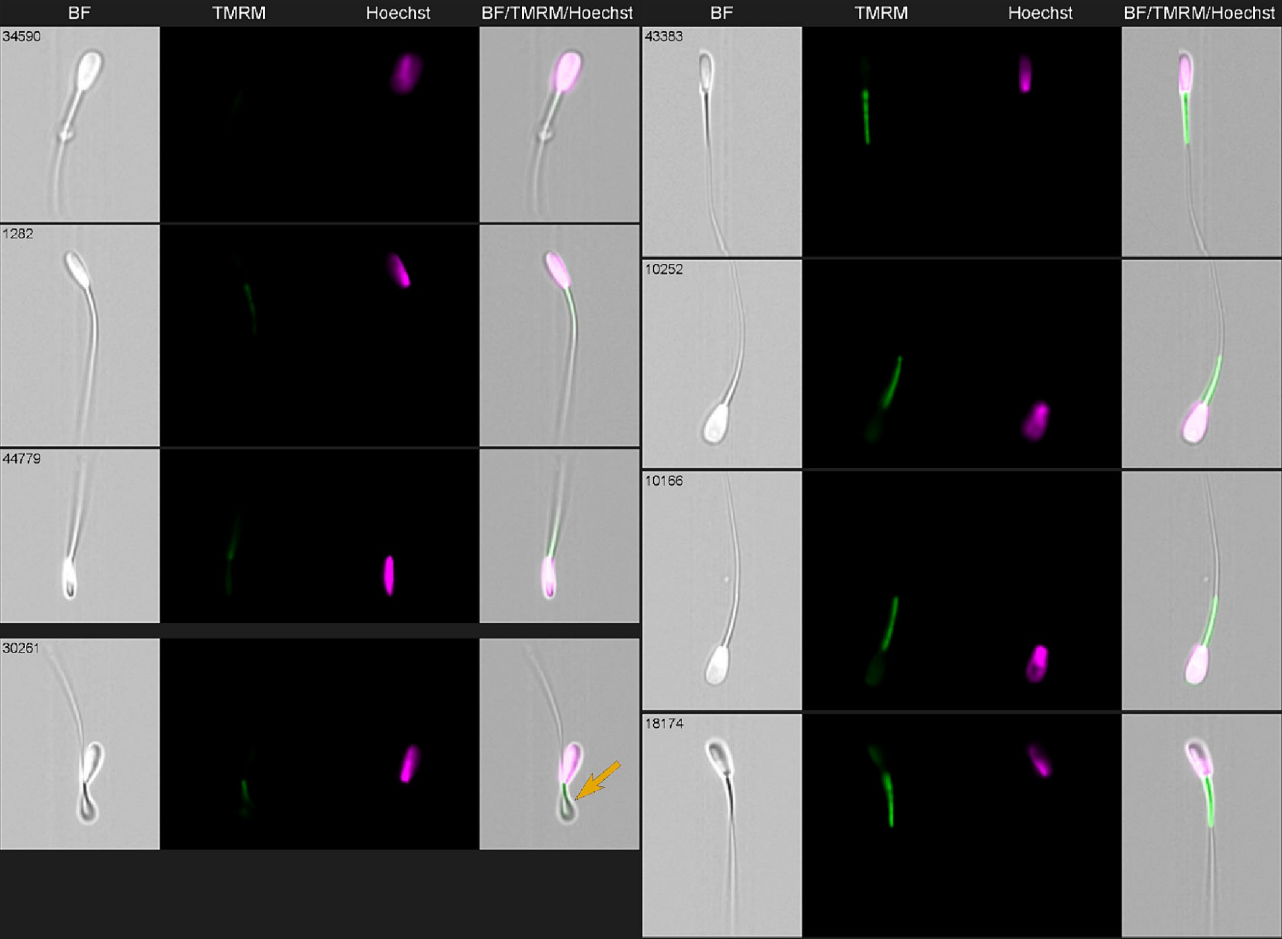
Imaging flow cytometry gallery of thawed bovine spermatozoa stained with Hoechst 33342 (green) and TMRM (purple) and acquired using ImageStream X Mark II (Amnis-Cytek, United States). Simultaneous observation of TMRM and Hoechst staining allows to differentiate normal (right row) and abnormal (left row) sperm. Top three sperm in the left—low TMRM with normal nuclei; lower left—one spermatozoa had no nucleus (arrow).

TMRE/TMRM mitochondrial staining is a specific marker of Δ*ψ*_m_ only when used at a low, non-quenching concentration (≤50 nM) (43). Below, Figure 6 provides representative images of the bovine spermatozoa stained with Hoechst 33342 for DNA (green) and TMRM dye for mitochondrial potential evaluation (purple). Evaluated morphological parameters included abnormal head, midpiece, tail, and so-called cytoplasmic “droplets” compared with morphologically normal cells (Figure 6). These morphological parameters are additive and can provide a percentage of morphologically and/or functionally aberrant spermatozoa (44).

## 4 Discussion

The growing interest in artificial insemination (AI) of cattle creates a need for more robust and intrinsic analyses of semen conditions. For a long time, morphological features of spermatozoa (45–47) and sperm motility (48) were used as the main quality parameters to evaluate. The morphological characteristics of healthy bovine spermatozoa include an oval-shaped head, lack of defects in the midpiece and tail, and absence of cytoplasmic droplets (17, 49, 50) and were observed mainly using light microscopy (17, 51, 52). However, in the last decade, parameters related to cellular metabolism such as plasma membrane integrity, mitochondrial potential, and DNA fragmentation index (DFI) of post-thawed sperm started to be used to evaluate semen quality and predict animal fertility (53–55). This led to broader use of flow cytometry and various fluorochromes for sperm evaluation (56). However, the flow cytometry analysis is complicated by the presence of autofluorescent “non-sperm events” originating from extender particles in sperm freezing medium, cellular or bacterial debris, and can lead to overestimating sperm populations and features (57).

Though the sperm parameters that define high fertilization potential are not fully understood, many studies indicate that sperm with defective parameters do not reach the place of fertilization.

Across the selected studies for this meta-analysis, the evaluation of thawed sperm quality parameters was mainly done on flow cytometers or microscopes. The total motility of sperm was analyzed on computer-assisted sperm analysis (CASA) for almost all studies. CASA-based systems have successfully evolved in sperm analysis over approximately 50 years measuring morphology and motility attributes of single sperm (58–60). However, interpretation of CASA data should be defined in terms of the measurement conditions, in particular, model and software version, time for tracking sample, microscope magnification and rate of image acquisition.

Some of the analyzed studies combined those methods to obtain a detailed quality assessment of bovine spermatozoa. Our meta-analysis focuses on the multiparametric evaluation of cryopreserved semen quality performed mainly through flow cytometry to characterize animal fertility. Cell viability is often taken as a control parameter of bovine sperm quality and fertilizing ability after thawing.

### 4.1 Sperm membrane damage and DNA fragmentation

When assessed with flow cytometry, sperm is usually stained with DNA fluorescent dyes such as propidium iodide (PI) (61), SYBR-14 (62, 63), or Hoechst 33342 (64) to evaluate sperm viability. Cells with damaged membranes allow PI to penetrate the cell and bind to DNA (22, 65, 66). The plasma membrane damage resulting from cryopreservation can at least in part be attributed to lipid peroxidation processes (67) and the generation of reactive oxygen species (ROS) (68). The membrane damage leads to a decline of the post-thawed bovine spermatozoa viability by an average of 50% (69, 70), which is consistent with the results obtained from our meta-analysis. Spermatozoa are viable when their plasma membrane is intact (71).

Sperm DNA fragmentation (SDF) is considered a suitable parameter for predicting fertility (72–75). DNA fragmentation index or DFI is simply a quantitative representation of SDF, calculated as a percentage of DNA fragmented sperm cells with DNA damage or abnormal protamination (74, 76, 77). Karoui and co-authors found a negative association between bull fertility and sperm DNA fragmentation using the halo test (78). DNA integrity is essential for bovine embryonic development (79), and DFI is negatively correlated with sperm binding index and conception rates (80). Interestingly, the sperm viability and motility become affected earlier than DNA, and occurrence of single-stranded DNA breaks happens in parallel with chromatin decondensation and deprotamination (81).

### 4.2 Sperm mitochondrial status

Mitochondrial status, another valuable metabolic parameter of sperm viability and, thus, fertility, is mainly characterized by mitochondrial membrane potential and shifts in mitochondrial functionality (56). The mitochondria are the “powerhouses” of the cell and are crucial for the proper motility of spermatozoa; therefore, monitoring changes in mitochondrial functionality is required to predict sperm fertility accurately (63, 82, 83). Moreover, mitochondria are highly prone to damage during cryopreservation, and any abnormalities in mitochondrial morphology or their functionality will decline sperm quality (84). Discrimination between high and low mitochondrial membrane potential is possible with different fluorescent dyes, including JC-1, rhodamine family dyes (rhodamine 123, TMRE, and TMRM), 3,3′-dihexiloxocarbocyanine iodide (DiOC6) and Mitotracker family dyes (85–88) and provides information about the quality of sperm (69, 84, 86, 89, 90). The uptake of most mitochondria-selective dyes, with some exceptions (acridine orange and Mitotracker Green), is dependent on ΔΨ (91). However, some of these dyes have properties that limit their use (88, 92). Thus, JC-1, a cationic dye that is actively used in sperm research, can only detect large differences in ΔΨ across cellular populations (93) and, therefore, subjected to artifacts (94, 95). Moreover, JC-1 double fluorescence made it challenging to use this dye combined with other fluorochromes (57) and its fluorescence can be affected by some components of the sperm freezing medium (94). A good alternative is TMRM dye, which accurately detects sperm populations displaying either high or low ΔΨ in the conditions where JC-1 has difficulties demonstrating differences (95). We also suggest TMRM staining as a mandatory MMP part of sperm quality evaluation.

Mitochondrial potential and DFI influence were assessed relative to the control viability parameter. DFI and abnormal morphology contribute the most to heterogeneity, suggesting inconsistency at the data sampling and analysis stages. However, reported MMP values suffer less from error and are more consistent across studies relative to spermatozoa viability. Furthermore, we found that MMP is positively associated with the total motility of bovine spermatozoa, proving that high mitochondrial potential values are related to higher total motility of the sperm.

We conclude that a combination of MMP and SDF values alone cannot confidently determine the viability of spermatozoa or be a substitution for evaluating other parameters like the motility of cryopreserved sperm. However, a combination of motility, MMP, and DFI might be a better indicator. Additionally, multiple regression analysis was conducted to analyze the general trend between the parameter variables. Among MMP and motility parameters, it was determined that an increase in the motility of spermatozoa has a more significant effect on viability.

### 4.3 IFC as a multi-parametric approach for sperm evaluation

As stated earlier, each minor alteration in MMP is essential in overall mitochondrial status, and flow cytometry is convenient for monitoring those changes (90). Therefore, a single complex multi-parametric technique, imaging flow cytometry, capable of substituting both flow cytometry and microscope, would be an appropriate option. As we conclude from this meta-analysis, a combination of morphological and fluorescent functional parameters may improve sperm quality evaluation. Morphometric sperm characteristics are between most important indicators of fertility (96) but are not available from conventional flow cytometry analysis. The imaging flow cytometry has already been successfully used in different laboratories for sperm evaluation (44, 97–100) and allowed the characterization of morphological and metabolic sperm parameters in thousands of spermatozoa simultaneously. The IFC is a relatively young technology that still needs to find a place in commercial applications and research, particularly in sperm characterization, where routine methods (sperm motility evaluation, etc.) have been established for decades. However, the Imagestream IDEAS software capable to differentiate sperm from cellular debris and cytoplasmic droplets of similar size solving the major problem of automatic CASA systems.

Artificial intelligence and machine learning algorithms are increasingly used to analyze IFC data (101–104). The development of IFC systems for this specific field will depend on reproducibility, usability, stability, complexity of instrument setup, and addition of artificial intelligence algorithms to remove the subjectivity and variability of analysis (105, 106). It requires improving the IFC systems to where data will be recorded, processed, and analyzed quickly in veterinary laboratories. Interestingly, current IFC systems (Imagestream and Flocyte instrument lines) are coming from a sole source (Amnis-Cytek) (107), which may simplify standardization of protocols. Recent introduction of image-based sorting instrumentation (108) capable to high-throughput sorting based on fluorescence and morphological features may prove helpful in sex-sorting of functional sperm.

### 4.4 Limitations of the study

First of all, the analyzed studies represented different breeds and sometimes a mix of breeds; after using subgroup analysis, the level of heterogeneity was decreased in the more homogenous dairy group of breeds. The number of studies used for each subgroup was relatively small that could result in a biased estimation of heterogeneity (*I*^2^) (109). Next, the lack of standardization contributed to the high heterogeneity level. The methods for evaluating total motility and abnormal morphology, thawing and staining techniques, and types of fluorescent dyes are varied among the studies due to the lack of standard protocols for quality assessment of cryopreserved bovine semen. Sperm morphology needs to be better evaluated, and though CASA systems have developed protocols to analyze efficiently the kinematic parameters of animal sperm (58), their use needs to be extended to the analysis of viability, DFI and morphology (110), which considered to be vital for fertility prediction.

## 5 Conclusion

In this study, cryopreserved sperm research exploring different post-thaw evaluation were compared. Results of the present meta-analysis indicate a significant positive MMP correlation with both total sperm motility and viability. The percentage of morphological abnormalities and DFI was lower in sperm with high viability. The MMP and motility were the most prominent sperm quality parameters, with minor publication bias and a notable association with sperm viability.

In conclusion, a combination of morphological assays, along with an evaluation of metabolic heterogeneity, including mitochondrial status, motility, and DNA integrity of sperm, is needed for sperm analysis standardization and a proper prediction of the fertilizing ability of sperm. Multi-parametric flow cytometry has become an important method for a rapid and sensitive evaluation of functional sperm properties and in veterinary science research as well as for a routine assessment of sperm quality. However, flow cytometry does not evaluate the morphological characteristics of semen. Furthermore, using a hybrid IFC technology which combines flow cytometry and light microscopy features, can potentially lead to solving one of the major problems in sperm evaluation, the inability to test morphological and functional parameters simultaneously, and will help with to standardize protocols for semen assessment. Future studies utilizing imaging flow cytometry for sperm evaluation may be combined with image-based sex-sorting of sperm.

## Data availability statement

The raw data supporting the conclusions of this article will be made available by the authors, without undue reservation.

## Author contributions

AU: Conceptualization, Data curation, Formal analysis, Investigation, Methodology, Project administration, Writing – original draft, Writing – review & editing. AK: Conceptualization, Data curation, Formal analysis, Investigation, Methodology, Writing – original draft, Writing – review & editing. AM: Formal analysis, Methodology, Writing – review & editing. BS: Conceptualization, Formal analysis, Supervision, Writing – review & editing. IV: Conceptualization, Funding, Supervision, Writing – review & editing. NB: Conceptualization, Funding acquisition, Methodology, Supervision, Validation, Writing – original draft, Writing – review & editing.
