# Supplementary material for: Systematic review and meta-analysis of cryopreserved bovine sperm assessment: harnessing imaging flow cytometry for multi-parametric analysis
Source: Front Vet Sci. 2024 Apr 24;11:1371586. doi: 10.3389/fvets.2024.1371586 (PMC11078101; doi:10.3389/fvets.2024.1371586)
Supplement: Supplementary file 1 [file Data_Sheet_1.docx]

**Systematic Review and Meta-Analysis of Cryopreserved Bovine Sperm Assessment: Harnessing Imaging Flow Cytometry for Multi-Parametric Analysis**

Anel Umirbaeva^1,#^, Andrey Kurenkov^2,#^, Aizhan Makhanbetova^3^, Bolat Seisenov^3^, Ivan A. Vorobjev^1^, Natasha S. Barteneva^1,^*

^1^Department of Biology, School of Sciences and Humanities, Nazarbayev University, Astana, 010000, Kazakhstan

^2^Department of Computer Sciences, School of Engineering and Digital Sciences, Nazarbayev University, 010000, Astana, Kazakhstan

^3^JSC“Republican Center of Breeding in Livestock” “Asyl-Tulik”, Akmola region, 100000, Kosshi, Kazakhstan

**^*^Corresponding author:**

**Natasha S. Barteneva,**

Department of Biology,

School of Sciences and Humanities,

Nazarbayev University

53 Kabanbay Batyr Avenue, 7ext522

Astana, 01000, Kazakhstan

e-mail: [natalie.barteneva@nu.edu.kz](mailto:natalie.barteneva@nu.edu.kz); bartene@yahoo.com

phone: +7(778)6357336

**List of abbreviations**

CASA – computer-assisted sperm analysis;

FC - flow cytometry

IFC - imaging flow cytometry

PI - propidium iodide

AO - acridine orange

SCSA - sperm chromatin structure assay

DIC microscopy - differential interference contrast microscopy


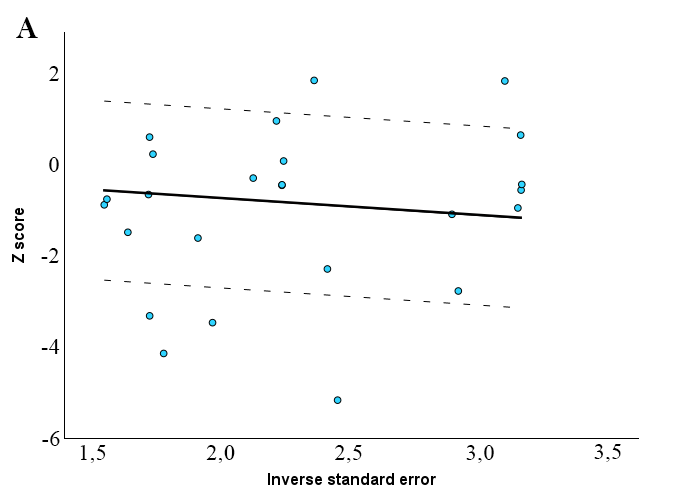


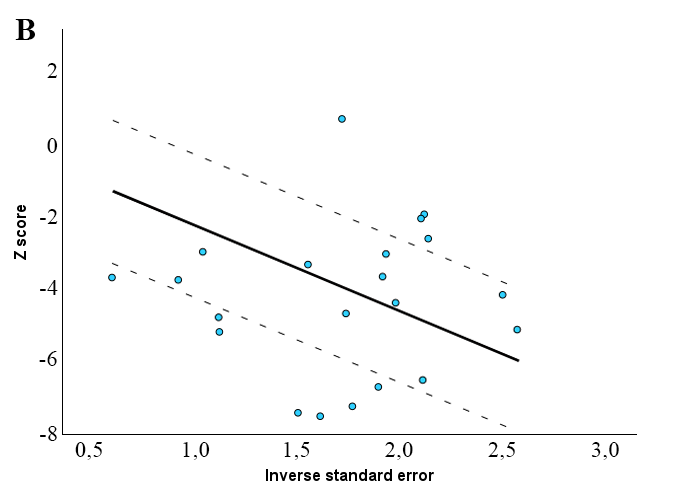


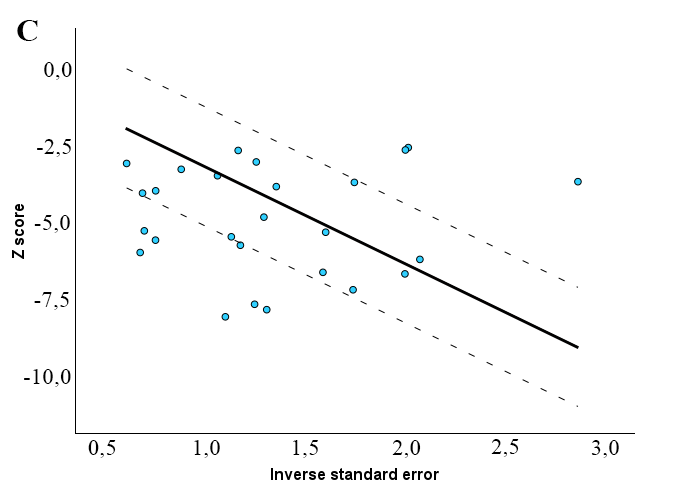


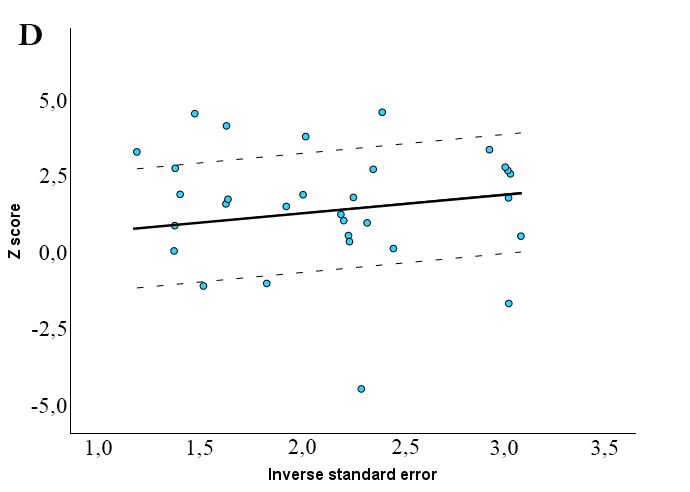


**Supplementary Figure S1.** Galbraith plot without outliers of the association between viability and MMP (**A**), Abnormal morphology (**B**), DFI (**C**), and Total motility (**D**)


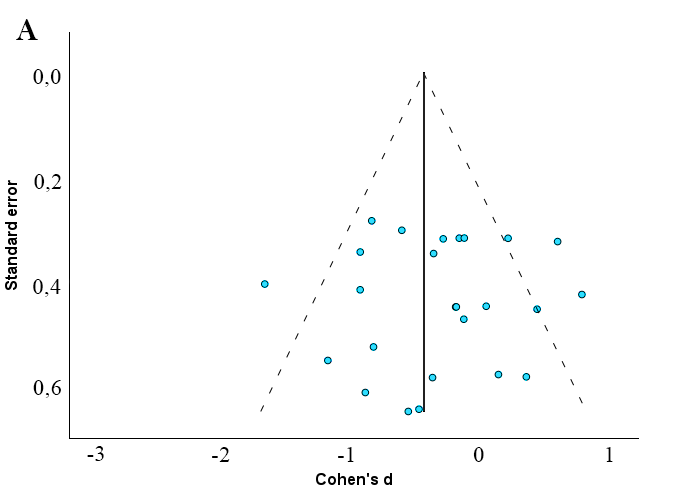


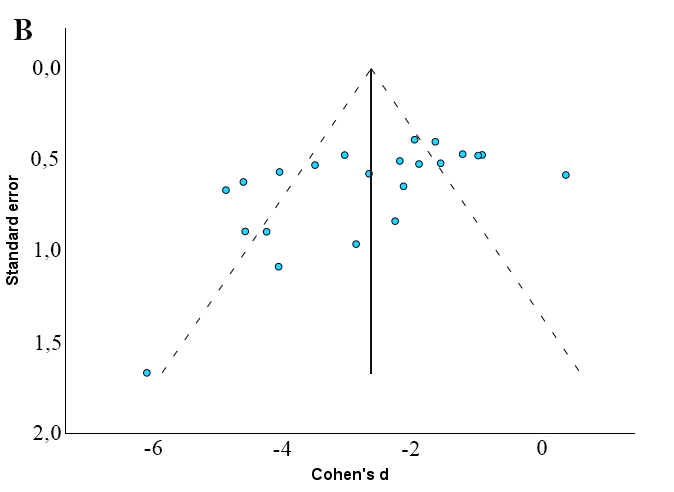


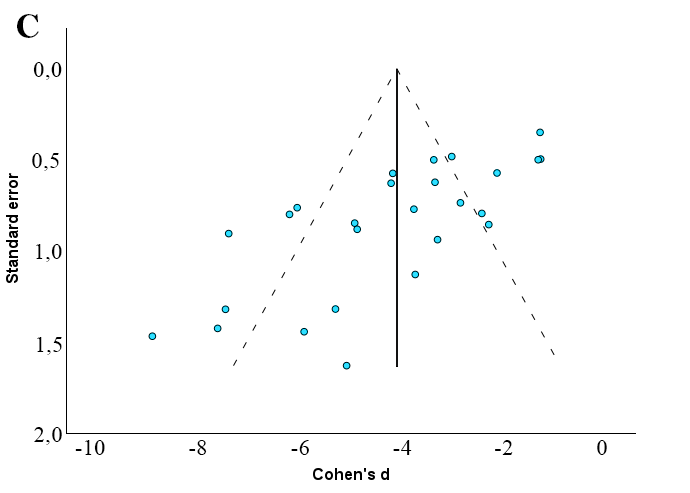


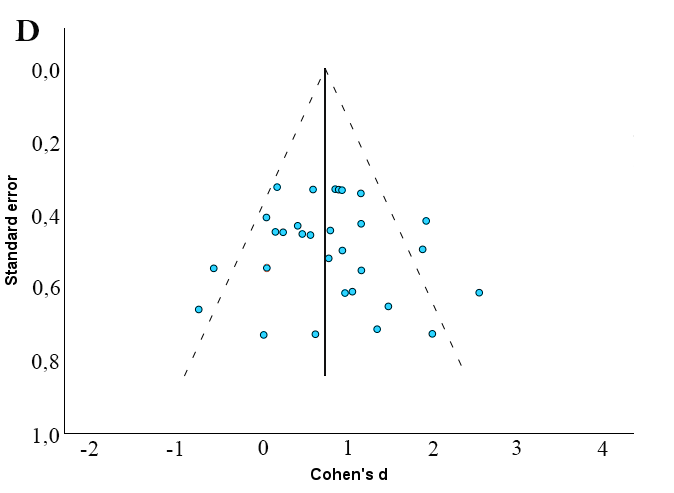


**Supplementary Figure S2.** Funnel plots without outliers depicting the presence of publication bias between included articles that determine the association between viability and MMP (**A**), Abnormal morphology (**B**), DFI (**C**), and Total motility (**D**).

**Table S1.** Descriptive characteristics of all selected studies.

| **#** | **Author** | **Year** | **Groups** | **Breed** | **Type** |
| --- | --- | --- | --- | --- | --- |
| 1 | Alm et al. | 2001 | Breed | Ayrshire, Holstein-Friesian | Dairy |
| 2 | Al Naib et al. | 2011 | Fertility | Holstein, Limousine | Dairy |
| 3 | Archana et al. | 2021 | Antigenicity  15 bulls- 38 samples | Holstein-Friesian | Dairy |
| 4 | Ballester et al. | 2007 | Drug treatment – 4 bulls | Swedish red | Dairy |
| 5 | Behnam et al. | 2023 | Extenders | Fleckvieh-Simmental | Dual |
| 6 | Bollwein et al. | 2008 | Thawing time | Simmental, Brown Swiss | Dairy |
| 7 | Carreira et al. | 2017 | Age | Bos Indicus:Nellore | Mix |
| 8 | Chaveiro et al. | 2015 | Before/after freezing | - | - |
| 9 | Esin et al. | 2022 | Thawing rate | Holstein | Dairy |
| 10 | Garcia-Macias et al. | 2007 | Fertility | Holstein-Friesian | Dairy |
| 11 | Gillan et al. | 2008 | Purification method | Holstein | Dairy |
| 12 | Gliozzi et al. | 2017 | Fertility | Holstein-Friesian | Dairy |
| 13 | Guo et al. | 2023 | Sex-sorting | Holstein Friesian | Dairy |
| 14 | Gurler et al. | 2016 | Freezing | Simmental, Brown Swiss | Dairy |
| 15 | Holden et al. | 2016 | Sex-sorting and thawing time | Holstein-Friesian | Dairy |
| 16 | Hurri et al. | 2022 | Ejaculates order | Holsten, Swedish red | Dairy |
| 17 | Jakop et al. | 2023 | Freezing characteristics | Holstein Friesian | Dairy |
| 18 | Januskauskas et al. | 2003 | Breeds-Swedish red and white | Swedish red and white | Dairy |
| 19 | Karoui et al. | 2012 | None | Holstein | Dairy |
| 20 | Khalil et al. | 2017 | Cryopreservation techniques | Friesian | Dairy |
| 21 | Kumaresan et al. | 2017 | Fertility | Swedish Red, Holstein | Dairy |
| 22 | Leite et al. | 2022 | Breed | Angus, Nellore | Beef/Mix |
| 23 | Malama et al. | 2017 | Season | Holstein-Friesian | Dairy |
| 24 | Morrell et al. | 2017 | Fertility | Holstein, Swedish Red, one each of Charolais, Limousin, Blonde, Svensk Kullig Boskap, | Dairy |
| 25 | Morrell et al. | 2018 | Breed | Beef bulls: Limousin, Charolais, Simmental, Hereford, Angus; Blonde  Diary: Swedish Red, Holstein | Dairy, Beef |
| 26 | Narud et al. | 2021 | Fertility | Norwegian Red | Dairy |
| 27 | Nongbua et al. | 2020 | Season | Bos indicus: American Brahman, Sahiwal | Beef/  Mix |
| 28 | Ntemka et al. | 2016 | Breed | Holstein, Brown Swiss, Limousin, Belgian Blue, Blonde d’Acquitante | Dairy |
| 29 | Okano et al. | 2019 | None | Nellore (Bos Indicus), Tropical Montana, Senepol, Holstein, Red Angus | Mix |
| 30 | Pardede et al. | 2023 | Post-thaw viability | Unknown, commercial semen straws | Unknown |
| 31 | Sabes-Alsina et al. | 2017 | Season | Holstein | Dairy |
| 32 | Sabes-Alsina et al. | 2019 | Season and countries | Holstein, Swedish Red | Dairy |
| 33 | Satrio et al. | 2022 | Age, semen rejection rate | Simmental | Dual |
| 34 | Sellem et al. | 2015 | Thawing time | Holstein | Dairy |
| 35 | Sobeh et al. | 2017 | Drug treatment | Friesian | Dairy |
| 36 | Takeda et al. | 2015 | Breed | Japanese Black (beef bull), Holstein | Beef, Dairy |
| 37 | Thomas et al. | 1997 | Thawing time | Holstein | Dairy |
| 38 | Thomas et al. | 1998 | Thawing time | Holstein | Dairy |
| 39 | Valeanu et al. | 2014 | Season | Swedish Red and White | Dairy |
| 40 | Zăhan et al. | 2018 | None | Fleckvieh-Simmental | Dual |

**Table S2.** Description of methods and stains used for semen assessment in each study.

| **#** | **Author** | **Year** | **DFI** | | **Morphology** | | **Viability** | | **MMP** | | **Motility** |
| --- | --- | --- | --- | --- | --- | --- | --- | --- | --- | --- | --- |
|  |  |  | **Method** | **Staining** | **Method** | **Staining** | **Method** | **Staining** | **Method** | **Staining** | **Method** |
| 1 | Alm et al. | 2001 | - | - | - | - | Fluorometric assay | PI | - | - | - |
| 2 | Alnaib et al. | 2011 | - | - | - | - | Fluorescent microscopy | SYBR-14/PI | - | - | - |
| 3 | Archana et al. | 2021 | - | - | - | - | Phase contrast microscopy | Eosin - nigrosin | Epifluorescence microscopy | JC-1 | - |
| 4 | Ballester et al. | 2007 | FC | AO | - | - | FC | SYBR-14/PI | FC | JC-1 | CASA |
| 5 | Benham et al. | 2023 | Light microscopy | Sperm DNA Fragmentation Assay Kit (SDFA; ACECR, Tehran, Iran) | Phase contrast microscopy | Hancock’s solution  (150 mL sodium saline solution, 150 mL buffer solution, 62.5 mL formalin (37%), and 500 mL double-distilled water) | Phase contrast microscopy | Hypoosmotic solution (1.9 mM sodium citrate and 5 mM fructose dissolved in 100 mL distilled water) | - | - | CASA |
| 6 | Bollwein et al. | 2008 | FC, SCSA | AO | - | - | FC | SYBR-14/PI | FC | JC-1 | - |
| 7 | Carreira et al. | 2017 | FC, SCSA | AO | DIC microscopy | - | FC | FITC-PSA/PI | FC | JC-1 | CASA |
| 8 | Chaveiro et al. | 2015 | - | - | - | - | FC | SYBR-14/PI | - | - | CASA |
| 9 | Esin et al. | 2022 | - | - | CASA | SpermBlue test kit (Microptic, Spain) | CASA | Eosin - nigrosin | - | - | CASA |
| 10 | Garcia-Maciaz et al. | 2007 | FC, SCSA | AO | Optical phase contrast microscopy | - | FC | SYBR-14/PI | FC | JC-1 | CASA |
| 11 | Gillan et al. | 2008 | - | - | Optical phase contrast microscopy | - | Fluorescent and phase contrast microscopy | Ethidium homodimer-1 (4.6 M final concentration, Molecular Probes Inc., Eugene, OR, USA) | - | - | CASA |
| 12 | Gliozzi et al. | 2017 | FC, SCSA | AO | - | - | FC | SYBR-14/PI | FC | JC-1 | CASA |
| 13 | Guo et al. | 2023 | - | - | - | - | - | - | - | - | CASA |
| 14 | Gurler et al. | 2016 | FC, SCSA | AO | - | - | FC | FITC-PNA/PI | FC | JC-1 | CASA |
| 15 | Holden et al. | 2016 | FC | AO | Phase contrast microscopy | Eosin - nigrosin | FC | PI | FC | JC-1 | Phase contrast microscopy |
| 16 | Hurri et al. | 2022 | FC | AO | - | - | FC | SYBR-14/PI | FC | JC-1 | CASA |
| 17 | Jakop et al. | 2023 | - | - | Phase contrast microscope | - | - | - | - | - | CASA |
| 18 | Januskaukus et al. | 2003 | - | - | - | - | Fluorometric assay | PI | - | - | - |
| 19 | Karoui et al. | 2012 | Light microscopy | Sperm–Halomax kit (Halotech DNA, Madrid, Spain). | - | - | - | - | - | - | Light microscopy |
| 20 | Khalil et al. | 2017 | - | - | Light microscopy | Eosin - nigrosin | Light microscopy | Eosin - nigrosin | - | - | Phase contrast microscopy |
| 21 | Kumaresan et al. | 2017 | FC, SCSA | AO | - | - | FC | SYBR-14/PI | - | - | - |
| 22 | Leite et al. | 2022 | - | - | Differential Interference Contrast Microscopy | - | FC | FITC-PSA/PI | - | - | CASA |
| 23 | Malama et al. | 2017 | FC, SCSA | AO | - | - | FC | FITC-PNA/PI | FC | JC-1 | CASA |
| 24 | Morell et al. | 2017 | FC, SCSA | AO | Light microscopy | William’s stain | FC | SYBR-14/PI | FC | JC-1 | CASA |
| 25 | Morell et al. | 2018 | FC, SCSA | AO | Light microscopy | Carbol fuchsin - eosin | FC | SYBR-14/PI | FC | JC-1 | CASA |
| 26 | Narud et al. | 2021 | FC, SCSA | AO | - | - | - | - | - | - | - |
| 27 | Nongbua et al. | 2020 | FC | AO | Phase contrast microscope | - | FC | SYBR-14/PI | FC | JC-1 | CASA |
| 28 | Ntemka et al. | 2016 | FC | AO | Light microscopy | Sperm Blue staining | Fluorescent light microscopy | PI/calcein | - | - | - |
| 29 | Okano et al. | 2019 | - | - | - | - | FC | FITC-PSA/PI | - | - | Optical microscopy |
| 30 | Pardede et al. | 2023 | - | - | - | - | FC | SYBR-14/PI | Fluorescent light microscopy | JC-1 | CASA |
| 31 | Sabes-Alsina et al. | 2017 | FC, SCSA | AO | Light microscopy | William's stain | FC | SYBR-14/PI | FC | JC-1 | CASA |
| 32 | Sabes-Alsina et al. | 2019 | FC, SCSA | AO | Light microscopy | William's stain | FC | SYBR-14/PI | FC | JC-1 | CASA |
| 33 | Satrio et al. | 2022 | Light microscopy | Sperm–Halomax kit (Halotech DNA, Madrid, Spain) | - | - | Light microscopy | Eosin - nigrosin | - | - | CASA |
| 34 | Sellem et al. | 2015 | - | - | IVOS II CASA system (Hamilton Thorne Inc.) | - | FC | EasyKit 1 Viability and Concentration (ref. 024708; IMV Technologies) | FC | EasyKit 2 (ref. 024864; IMV Technologies) | CASA |
| 35 | Sobeh et al. | 2017 | - | - | Light microscopy | Eosin - nigrosin | Light microscopy | Eosin - nigrosin | - | - | Phase contrast microscopy |
| 36 | Takeda et al. | 2015 | FC | Tunnel assay kit (In situ Cell Death Detection Kit, fluorescein, Roche, Indianapolis, IN, USA) | - | - | - | - | - | - | - |
| 37 | Thomas et al. | 1997 | - | - | - | - | FC | SYBR-14/PI | - | - | - |
| 38 | Thomas et al. | 1998 | - | - | - | - | FC | SYBR-14/PI | FC | JC | Light microscopy |
| 39 | Valeanu et al. | 2014 | FC, SCSA | AO | Light microscopy | Carbol fuchsin - eosin | FC | SYBR-14/PI | FC | JC | CASA |
| 40 | Zahan et al. | 2018 | Bright-field microscopy | Bovine-Halomax kit (ChromaCell SL, Madrid, Spain) | - | - | FC | SYBR-14/PI | FC | JC-1 | CASA |

**References:**

1. Alm K, Taponen J, Dahlbom M, Tuunainen E, Koskinen E, Andersson M. A novel automated fluorometric assay to evaluate sperm viability and fertility in dairy bulls. Theriogenology. 2001 Sep; 56(4):677–84. doi: [10.1016/s0093-691x(01)00599-4](https://doi.org/10.1016/s0093-691x(01)00599-4) PMID: 11572448
2. Al Naib A, Hanrahan JP, Lonergan P, Fair S. In vitro assessment of sperm from bulls of high and low field fertility. Theriogenology. 2011 Jul; 76(1):161–7. doi: [10.1016/j.theriogenology.2010.10.038](https://doi.org/10.1016/j.theriogenology.2010.10.038) PMID: 21396687
3. Archana SS, Selvaraju S, Arangasamy A, Binsila B, Swathi D, Ramya L, et al. Seminal antigenicity affects mitochondrial membrane potential and acrosome reaction ability of the spermatozoa during cryopreservation. Theriogenology. 2021 Jan 1; 159:132–9. doi: [10.1016/j.theriogenology.2020.10.025](https://doi.org/10.1016/j.theriogenology.2020.10.025) PMID: 33137634
4. Ballester J, Johannisson A, Saravia F, Håård M, Gustafsson H, Bajramovic D, et al. Post-thaw viability of bull AI-doses with low-sperm numbers. Theriogenology. 2007 Oct 1; 68(6):934–43. doi: [10.1016/j.theriogenology.2007.07.008](https://doi.org/10.1016/j.theriogenology.2007.07.008) PMID: 17707900
5. Behnam M, Asadpour R, Topraggaleh TR, Hamali H. Improvement of post-thaw quality and fertilizing ability of bull spermatozoa using rho kinase inhibitor in freezing extender. Frontiers in Veterinary Science. 2023;10. doi:10.3389/fvets.2023.1155048
6. Bollwein H, Fuchs I, Koess C. Interrelationship between plasma membrane integrity, mitochondrial membrane potential and DNA fragmentation in cryopreserved bovine Spermatozoa. Reprod Domest Anim. 2008 Apr; 43(2):189–95. doi: [10.1111/j.1439-0531.2007.00876.x](https://doi.org/10.1111/j.1439-0531.2007.00876.x) PMID: 17986172
7. Carreira JT, Trevizan JT, Carvalho IR, Kipper B, Rodrigues LH, Silva C, et al. Does sperm quality and DNA integrity differ in cryopreserved semen samples from young, adult, and aged Nellore bulls? Basic Clin Androl. 2017 Jun 21; 27:12. doi: [10.1186/s12610-017-0056-9](https://doi.org/10.1186/s12610-017-0056-9) PMID: 28649382
8. Chaveiro A, Cerqueira C, Silva J, Franco J, Moreira da Silva F. Evaluation of frozen thawed cauda epididymal sperms and in vitro fertilizing potential of bovine sperm collected from the cauda epididymal. Iran J Vet Res. 2015 Spring; 16(2):188–93. PMID: 27175174
9. Esin B, Akar M, Tagrikulu MD, Kaya C, Çevik M. The effects of fast and slow thawing on spermatological parameters and detect of chromatin condensation by toluidine blue staining in frozen-thawed bull sperm. Kafkas Univ Vet Fak Derg. 2022 May; 28(3):307–13. doi: [10.9775/kvfd.2021.26950](http://dx.doi.org/10.9775/kvfd.2021.26950)
10. García-Macías V, de Paz P, Martinez-Pastor F, Álvarez M, Gomes-Alves S, Bernardo J, et al. DNA fragmentation assessment by flow cytometry and Sperm-Bos-Halomax (bright-field microscopy and fluorescence microscopy) in bull sperm. Int J Androl. 2007 Apr; 30(2):88–98. doi: [10.1111/j.1365-2605.2006.00723.x](https://doi.org/10.1111/j.1365-2605.2006.00723.x) PMID: 17166172
11. Gillan L, Kroetsch T, Chis Maxwell WM, Evans G. Assessment of in vitro sperm characteristics in relation to fertility in dairy bulls. Anim Reprod Sci. 2008 Jan; 103(3-4):201–14. doi: [10.1016/j.anireprosci.2006.12.010](https://doi.org/10.1016/j.anireprosci.2006.12.010) PMID: 17208395
12. Gliozzi TM, Turri F, Manes S, Cassinelli C, Pizzi F. The combination of kinetic and flow cytometric semen parameters as a tool to predict fertility in cryopreserved bull semen. Animal. 2017 Nov; 11(11):1975–82. doi: [10.1017/S1751731117000684](https://doi.org/10.1017/s1751731117000684) PMID: 28397643
13. Guo Y, Fan Z, Zhao F, Ge S, Chu H, Wei Z, et al. Assessment of Semen Quality and anti‐oxidative enzyme activity between bovine sex‐sorted and non‐sex‐sorted frozen–thawed semen. Reproduction in Domestic Animals. 2023;58(5):657–61. doi:10.1111/rda.14333
14. Gürler H, Malama E, Heppelmann M, Calisici O, Leiding C, Kastelic JP, et al. Effects of cryopreservation on sperm viability, synthesis of reactive oxygen species, and DNA damage of bovine sperm. Theriogenology. 2016 Jul; 86(2):562–71. doi: [10.1016/j.theriogenology.2016.02.007](https://doi.org/10.1016/j.theriogenology.2016.02.007) PMID: 27039074
15. Holden SA, Fernandez-Fuertes B, Murphy C, Whelan H, O'Gorman A, Brennan L, Butler ST, Lonergan P, Fair S. Relationship between in vitro sperm functional assessments, seminal plasma composition, and field fertility after AI with either non-sorted or sex-sorted bull semen. Theriogenology. 2017 Jan 1;87:221-228. doi: 10.1016/j.theriogenology.2016.08.024. Epub 2016 Sep 3. PMID: 27678515.
16. Hurri E, Lima‐Verde I, Johannisson A, Stålhammar H, Ntallaris T, Morrell JM. Post‐thaw semen quality in young bull ejaculates before being accepted for commercial semen doses. Vet Rec. 2022 Sep; 191(6):e1386. doi: [10.1002/vetr.1386](https://doi.org/10.1002/vetr.1386) PMID: 35137420
17. Jakop U, Engel KM, Hürland M, Müller P, Osmers J-H, Jung M, et al. Lipid alterations by oxidative stress increase detached acrosomes after cryopreservation of semen in Holstein bulls. Theriogenology. 2023;197:37–45. doi:10.1016/j.theriogenology.2022.11.036
18. Januskauskas A, Johannisson A, Rodriguez-Martinez H. Subtle membrane changes in cryopreserved bull semen in relation with sperm viability, chromatin structure, and field fertility. Theriogenology. 2003 Sep; 60(4):743–58. doi: [10.1016/s0093-691x(03)00050-5](https://doi.org/10.1016/s0093-691x(03)00050-5) PMID: 12832022
19. Karoui S, Díaz C, González-Marín C, Amenabar ME, Serrano M, Ugarte E, et al. Is sperm DNA fragmentation a good marker for field AI bull fertility? J Anim Sci. 2012 Aug; 90(8):2437–49. doi: [10.2527/jas.2011-4492](https://doi.org/10.2527/jas.2011-4492) PMID: 22367070
20. Khalil WA, El-Harairy MA, Zeidan AEB, Hassan MAE, Mohey-Elsaeed O. Evaluation of bull spermatozoa during and after cryopreservation: Structural and ultrastructural insights. Int J Vet Sci Med. 2017 Nov; 6(supp1):S49–56. doi: [10.1016/j.ijvsm.2017.11.001](https://doi.org/10.1016/j.ijvsm.2017.11.001) PMID: 30761321
21. Kumaresan A, Johannisson A, Al-Essawe EM, Morrell JM. Sperm viability, reactive oxygen species, and DNA fragmentation index combined can discriminate between above- and below-average fertility bulls. J Dairy Sci. 2017 Jul; 100(7):5824–36. doi: [10.3168/jds.2016-12484](https://doi.org/10.3168/jds.2016-12484) PMID: 28478003
22. Leite RF, Losano JDA, Kawai GKV, Rui BR, Nagai KK, Castiglioni VC, Siqueira AFP, D'Avila Assumpção MEO, Baruselli PS, Nichi M. Sperm function and oxidative status: effect on fertility in Bos taurus and Bos indicus bulls when semen is used for fixed-time artificial insemination. Anim Reprod Sci. 2022 Feb; 237:106922. doi: 10.1016/j.anireprosci.2022.106922. PMID: 35065462
23. Malama E, Zeron Y, Janett F, Siuda M, Roth Z, Bollwein H. Use of computer-assisted sperm analysis and flow cytometry to detect seasonal variations of bovine semen quality. Theriogenology. 2017 Jan; 87:79–90. doi: [10.1016/j.theriogenology.2016.08.002](https://doi.org/10.1016/j.theriogenology.2016.08.002) PMID: 27720270
24. Morrell JM, Nongbua T, Valeanu S, Lima Verde I, Lundstedt-Enkel K, Edman A, et al. Sperm quality variables as indicators of bull fertility may be breed dependent. Anim Reprod Sci. 2017 Oct; 185:42–52. doi: [10.1016/j.anireprosci.2017.08.001](https://doi.org/10.1016/j.anireprosci.2017.08.001) PMID: 28811063
25. Morrell JM, Valeanu AS, Lundeheim N, Johannisson A. Sperm quality in frozen beef and dairy bull semen. Acta Vet Scand. 2018 Jul 4; 60(1):41. doi: [10.1186/s13028-018-0396-2](https://doi.org/10.1186/s13028-018-0396-2) PMID: 29973236
26. Narud B, Khezri A, Zeremichael TT, Stenseth EB, Heringstad B, Johannisson A, et al. Sperm chromatin integrity and DNA methylation in Norwegian Red bulls of contrasting fertility. Mol Reprod Dev. 2021 Mar; 88(3):187–200. doi: [10.1002/mrd.23461](https://doi.org/10.1002/mrd.23461) PMID: 33634579
27. Nongbua T, Utta A, Am-in N, Suwimonteerabutr J, Johannisson A, Morrell JM. Effects of season and single layer centrifugation on bull sperm quality in Thailand. Asian-Australasian J Anim Sci. 2020 Sep; 33(9):1411–20. doi: [10.5713/ajas.19.0624](https://doi.org/10.5713/ajas.19.0624) PMID: 32054188
28. Ntemka A, Tsousis G, Brozos C, Kiossis E, Boscos C, Tsakmakidis I. Breed differences of bull frozen-thawed semen. Reprod Domest Anim. 2016 Dec; 51(6):945–52. doi: [10.1111/rda.12769](https://doi.org/10.1111/rda.12769) PMID: 27667130
29. Okano DS, Penitente-Filho JM, Gomez León VE, Maitan PP, Silveira CO, Waddington B, et al. In vitro evaluation of cryopreserved bovine sperm and its relation to field fertility in fixed-time artificial insemination. Reprod Domest Anim. 2019 Mar; 54(3):604-612. doi: 10.1111/rda.13401 PMID: 30614080
30. Pardede BP, Kusumawati A, Pangestu M, Purwantara B. Bovine sperm HSP-70 molecules: A potential cryo-tolerance marker associated with semen quality and fertility rate. Frontiers in Veterinary Science. 2023;10. doi:10.3389/fvets.2023.1167594
31. Sabés-Alsina M, Johannisson A, Lundeheim N, Lopez-Bejar M, Morrell JM. Effects of season on bull sperm quality in thawed samples in northern Spain. Vet Rec. 2017 Mar 11; 180(10):251. doi: [10.1136/vr.103897](https://doi.org/10.1136/vr.103897) PMID: 28100767
32. Sabés-Alsina M, Lundeheim N, Johannisson A, López-Béjar M, Morrell JM. Relationships between climate and sperm quality in dairy bull semen: a retrospective analysis. J Dairy Sci. 2019 Jun; 102(6):5623–33. doi: [10.3168/jds.2018-15837](https://doi.org/10.3168/jds.2018-15837) PMID: 30904295
33. Satrio FA, Karja NW, Setiadi MA, Kaiin EM, Gunawan M, Purwantara B. Post-thaw characteristics of the Simmental sperm function in different ages of bulls. Tropical Animal Science Journal. 2022;45(4):381–8. doi:10.5398/tasj.2022.45.4.381
34. Sellem E, Broekhuijse MLWJ, Chevrier L, Camugli S, Schmitt E, Schibler L, et al. Use of combinations of in vitro quality assessments to predict fertility of bovine semen. Theriogenology. 2015 Dec; 84(9):1447-1454.e5. doi: [10.1016/j.theriogenology.2015.07.035](https://doi.org/10.1016/j.theriogenology.2015.07.035) PMID: 26296523
35. Sobeh M, Hassan S, El Raey M, Khalil W, Hassan M, Wink M. Polyphenolics from Albizia harveyi exhibit antioxidant activities and counteract oxidative damage and ultra-structural changes of cryopreserved bull semen. Molecules. 2017 Nov 17; 22(11):1993. doi: [10.3390/molecules22111993](https://doi.org/10.3390/molecules22111993) PMID: 29149062
36. Takeda K, Uchiyama K, Kinukawa M, Tagami T, Kaneda M, Watanabe S. Evaluation of sperm DNA damage in bulls by TUNEL assay as a parameter of semen quality. J Reprod Dev. 2015; 61(3):185–90. doi: [10.1262/jrd.2014-140](https://doi.org/10.1262/jrd.2014-140) PMID: 25739957
37. Thomas CA, Garner DL, Mel DeJarnette J, Marshall CE. Fluorometric assessments of acrosomal integrity and viability in cryopreserved bovine spermatozoa. Biol Reprod. 1997 Apr; 56(4):991–8. doi: [10.1095/biolreprod56.4.991](https://doi.org/10.1095/biolreprod56.4.991) PMID: 9096883
38. Thomas CA, Garner DL, DeJarnette JM, Marshall CE. Effect of cryopreservation on bovine sperm organelle function and viability as determined by flow cytometry. Biol Reprod. 1998 Mar; 58(3):786–93. doi: [10.1095/biolreprod58.3.786](https://doi.org/10.1095/biolreprod58.3.786) PMID: 9510967
39. Valeanu S, Johannisson A, Lundeheim N, Morrell JM. Seasonal variation in sperm quality parameters in Swedish red dairy bulls used for artificial insemination. Livestock Science. 2015 Mar; 173:111–8. doi: [10.1016/j.livsci.2014.12.005](https://doi.org/10.1016/j.livsci.2014.12.005)
40. Zăhan M, Pall E, Cenariu M, Miclea I, Dascăl A. Relationship between in vitro semen parameters and bull fertility. ABAH Bioflux. 2018; 10(2):156-63
